# Letter from Dr. Berry

**Published:** 1848-04

**Authors:** Archibald Berry


### LETTER FROM DR. BERRY.

Prof. Taylor:

*Dear Sir*:—In compliance with my promise to transmit any thing of interest occurring in my visit to the South, I send you a hastily written report of two cases of the loss of healthy teeth, consequent upon pain produced by others that were decayed.

*Case* I.—Mr. W., aged about 35 years, in good health, was recently attacked with pain in a carious inferior dens sapientiæ, and also in the posterior superior bicuspid of the same side. The pain was experienced only at night, and then it was almost constant. Holding cold water in the mouth afforded temporary relief, and he kept it by his bed side almost every night for two weeks. The pain in the bicuspid became so severe that he resolved to endure it no longer, and had a gentleman not accustomed to extracting teeth to remove it with a small pair of pincers. As the instrument was not adapted to the purpose, in extracting it, about one-fourth of the crown of the anterior bicuspid was broken off. The pain in the socket continued for three weeks as before the tooth was extracted. When hearing from the gentleman a history of the case, I told him the pain in the bicuspid and its socket was undoubtedly caused by the dens sapientiæ, which still continued to ache at night. Having extracted it, there was no recurrence of the pain, its cause being evidently removed.

On examining the bicuspid that was extracted, it was apparently in a healthy condition at the time of its removal, although it had been filled five or six years previously. Had a correct diagnosis been made it could probably have been preserved for many years.

*Case* II.—Dr. ———, of this county, with a fine set of teeth, as he supposed free from caries, was several years ago seized with a severe pain in a superior molar. He consulted two dentists and several physicians, none of whom could discover the cause of the pain, which had continued severe for a considerable time. At length the aching tooth was extracted; but the pain in its socket continued as before. After some time had elapsed, he discovered the antagonist of the molar that had been removed, somewhat decayed; and, although it had never ached, he had it extracted. The pain ceased immediately, and did not occur again.

Respectfully yours, ARCHIBALD BERRY.

*Hinds County, Miss., Dec.* 18, 1847.

